# Pineal gland dysfunction in Alzheimer’s disease: relationship with the immune-pineal axis, sleep disturbance, and neurogenesis

**DOI:** 10.1186/s13024-019-0330-8

**Published:** 2019-07-11

**Authors:** Juhyun Song

**Affiliations:** Department of Anatomy, Chonnam National University Medical School, Hwasun, 58128 Jeollanam-do Republic of Korea

**Keywords:** Pineal gland, Pineal calcification, Alzheimer’s disease (AD), Circadian rhythms, Melatonin, Immune-pineal axis

## Abstract

Alzheimer’s disease (AD) is a globally common neurodegenerative disease, which is accompanied by alterations to various lifestyle patterns, such as sleep disturbance. The pineal gland is the primary endocrine organ that secretes hormones, such as melatonin, and controls the circadian rhythms. The decrease in pineal gland volume and pineal calcification leads to the reduction of melatonin production. Melatonin has been reported to have multiple roles in the central nervous system (CNS), including improving neurogenesis and synaptic plasticity, suppressing neuroinflammation, enhancing memory function, and protecting against oxidative stress. Recently, reduced pineal gland volume and pineal calcification, accompanied by cognitive decline and sleep disturbances have been observed in AD patients. Here, I review current significant evidence of the contribution of pineal dysfunction in AD to the progress of AD neuropathology. I suggest new insights to understanding the relationship between AD pathogenesis and pineal gland function.

## Background

Alzheimer’s disease (AD) is the most common neurodegenerative disease and is characterized by progressive loss of memory function and other neurobehavioral manifestations [1, 2]. The pathological hallmarks of AD have been reported, including extracellular senile plaques, which are mainly composed of β-amyloid (Aβ) and intracellular neurofibrillary tangles (NFTs) [1]. Current research reported that at present there are more than 47 million AD patients globally, and that this number will be projected to triple to nearly 150 million by 2050 [3]. The development of AD is accompanied by changes in lifestyle factors, such as sleep disturbance [3]. Unlike other neurodegenerative diseases, AD patients show sleep disturbances from an early phase [4]. Several studies suggest that the sleep disturbance in AD is an important diagnostic indicator for predicting AD progress [5, 6]. Due to this evidence, the relationship between pineal gland dysfunction and AD neuropathology is emerging as a new concept in understanding AD pathology, and suggests that circadian rhythms that control sleep disturbances are regulated by the pineal gland [7].

The pineal gland is a circumventricular organ that is derived from the embryonic forebrain, and it is the major part of the epithalamus, along with the habenular nuclei [7]. The pineal gland has been reported to secrete melatonin and directly control circadian rhythms in humans [8]. Melatonin is the main hormone produced by pineal gland, and is known to involved in antioxidant defense, immune responses, neuroprotective effects, anti-amyloid effects, and anti-apoptotic activity [9, 10]. Recently, studies have found lower levels of melatonin in AD, and noted that the decreased melatonin secretion triggers cognitive impairment [8, 11, 12]. The secretory capacity of the pineal gland is directly proportional to the pineal parenchymal volume and pineal gland function [13, 14]. Pineal calcification, also referred to as “brain sand”, is caused by hydroxyapatite deposition in the pineal gland [15, 16]. Certain studies have reported the reduced pineal volume and have found calcification in AD [17, 18]. Even though these relationship between pineal gland function and AD neuropathology were markedly found through various researches, the importance about that has not been highlighted until recent years.

Here, I review the recent evidences that how pineal dysfunction, by pineal volume reduction and pineal calcification, is involved in the AD pathogenesis.

### Pineal gland dysfunction in Alzheimer’s disease

Pineal gland is an endocrine organ localized in the human brain, and is present in a variety of weights and sizes among individuals [19]. Several studies demonstrated that the morphology and function of pineal gland are influenced by various physiological conditions [20]. Generally, the pineal gland has been reported to synthesize and secrete melatonin as a neuroendocrine hormone, which can regulate circadian rhythms in humans [21, 22]. In order to produce melatonin, the transcription of aralkylamine N-acetyltransferase (*Aanat)* and phosphorylation of AANAT are controlled on a daily basis by the pineal gland, and its activity is modulated by photoperiod seasonal change [23]. Furthermore, the phosphorylation of AANAT by protein kinase A (PKA) is mediated by the stimulation of pinealocytes, and ultimately, contributes to the production of melatonin [24]. The pineal gland consists primarily of pinealocytes, a few microglia, and astrocytes [25]. A portion of the pineal gland is exposed into the cerebrospinal fluid (CSF) of the third ventricle [26]. A large number of canaliculi of the pineal gland open directly into the CSF of the third ventricle, resulting in high melatonin level in the CSF of the third ventricle [27, 28].

Melatonin production is directly controlled by the internal circadian timer, which is located in the suprachiasmatic nucleus (SCN) [29] and also known as the “pacemaker” [30]. Membrane receptors of melatonin have been identified in the SCN [31, 32], and the signal transduction pathways through melatonin receptor 1 and 2 (MT1 and MT2) increase the expression of clock genes, including *Period circadian regulator 1* (Per1) [33, 34]. Therefore, the melatonin action through melatonin receptors contributes to the circadian rhythms. The volume of the pineal gland is correlated with the function of the pineal gland, because pinealocytes which produce melatonin mainly compose pineal gland. One study demonstrated that the volume of the pineal gland is also significantly reduced in patients with insomnia, and noted that lower pineal gland volumes contribute to sleep disorders [18].

In AD patients, the level of melatonin in CSF and blood serum was decreased compared to normal subjects and decreased level of melatonin finally leads to the aberrant diurnal rhythm [35–37]. Some studies demonstrated that reduced level of melatonin in AD brain contributes to the cognitive decline in AD patients and also is linked to the pineal gland volume [35, 37, 38]. Furthermore, the expression of melatonin receptor such as MT2 was decreased in the hippocampus of AD patients [39, 40]. The level of melatonin was observed the reduction of it in compared with normal subjects and also the aberrant size of pineal gland was found in AD patients. As mentioned, the reduction of melatonin level is important feature in AD patients. Some actions of melatonin in AD have been reported by various researchers. In AD, melatonin could efficiently inhibit tau hyperphosphorylation [41] and attenuate levels of secreted soluble amyloid beta precursor protein (APP) from neurons [42]. Melatonin administration attenuated Aβ generation and deposition in AD mice [43, 44]. Moreover, melatonin suppressed the peroxynitrite-induced inhibition of choline transport in neuronal proteins from synaptosomes and synaptic vesicles [45]. In AD, melatonin attenuated the accumulation of Aβ plaques that trigger pro-inflammatory responses and oxidative stress in the brain, causing cognitive impairment [46, 47]. Therefore, melatonin action may be critical to improve neuropathogenesis in AD based on upper findings.

Even though melatonin is secreted in pineal glands, the amount of melatonin synthesized by extrapineal organs was greater than the amount of melatonin secreted by the pineal gland [48]. However, the melatonin synthesized by extrapineal organs could not replace the function of melatonin produced by pineal gland, such as the regulation of circadian rhythms [49], the neuroprotection, and anti-inflammatory responses [50, 51]. Therefore, the melatonin produced by the pineal gland is important and irreplaceable in suppressing neuropathogenesis in AD brains. Regarding previous consequences, the dysfunction of pineal gland on neuropathology is an important issue to be investigated in AD brain.

Pineal calcification is calcium deposition in pineal gland, which has long been reported in humans [52, 53]. The occurrence of pineal calcification depends on environmental factors, such as sunlight exposure [54], and results in the decrease of melatonin production [55, 56]. Pineal calcification occurs when calcareous deposits form within the connective tissue of the pineal gland stroma, and it is similar to calcification observed in the habenular commissure and choroid plexus [57]. Unlike kidney stones, the main component of pineal calcification is hydroxyapatite [Ca_10_(PO_4_)_6_(OH)_2_], and the Ca/P molar ratio in pineal calcification is similar to that found in the enamel and dentine of teeth [58]. Morphological changes associated with pineal calcification include changes in the production of melatonin, due to the decreased function in the pineal gland parenchyma, and results in decreased pineal volume, reduced melatonin production in humans [14], and altered sleep patterns [59]. A few studies reported that pineal calcification [13, 14] and pineal cysts [60] trigger severe sleep disorders by disturbing melatonin secretion in the pineal gland. In clinical studies, patients with primary insomnia showed reduced plasma melatonin levels during the daytime [61].

Considering some studies, pineal calcification contributes to the reduction of melatonin production in humans that is directly associated with the development of neurodegenerative diseases, such as AD [54, 56, 62]. Previous researches demonstrated that the reduction of melatonin levels in CSF and serum leads to the aggravation of AD neuropathology [37, 38, 63]. In AD, reduced pineal size, pineal gland dysfunction, and pineal calcification have been reported [38], and decreased melatonin levels have been detected in serum [64] and urine [65]. A recent computed tomography study clearly observed pineal calcification in AD patients [56].

**Fig. 1:**
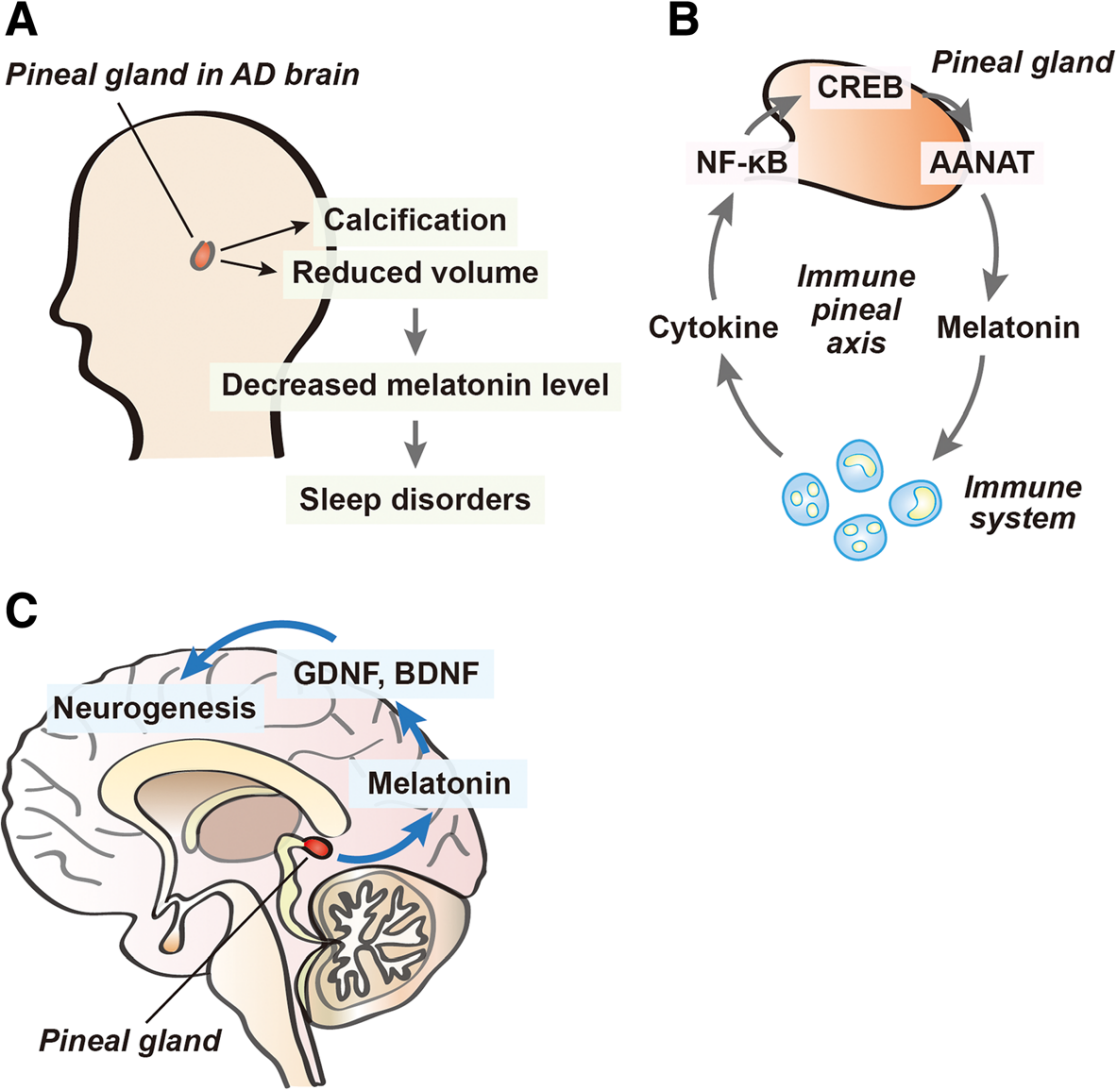
The schematic diagram of pineal gland dysfunction in AD, immune-pineal axis, and the role of melatonin in neurogenesis. **a** Pineal calcification and reduced pineal volume causes pineal dysfunction, which are commonly observed in AD brain. The pineal dysfunction leads to the reduction of melatonin level, subsequently results in sleep deficit. **b** Immune cells could regulate NF-κB activation and promote the production of melatonin in pinealocytes through CREB-AANAT signaling. **c** The decreased melatonin level leads to the impairment of neurogenesis in AD, because reduced melatonin level contributes the reduction of BDNF and GDNF expression, which are known as boosters in neurogenesis

Regarding these observations, pineal dysfunction reduces melatonin production, and, ultimately, contributes to diverse AD neuropathologies (Fig. 1a). However, the detailed mechanisms on pineal gland calcification and pineal gland dysfunction in AD are not fully understood yet. The mechanism related with pineal gland dysfunction in AD should be investigated to find therapeutic solution to cure AD pathologies, given that the pineal gland dysfunction is strongly linked to the AD pathologies.

### Impaired immune-pineal axis by pineal gland dysfunction and AD

The pineal gland, as part of the circumventricular organ, interacts with various molecules present in the blood and CSF [66]. Inflammatory mediators, such as cytokines, regulate the function of the pineal gland, leading to the suppression (i.e., secretion of proinflammatory cytokines) or potentiation (i.e., secretion of glucocorticoids) of melatonin synthesis [67]. Melatonin, derived from serotonin (5-HT), is synthesized in a rhythmic manner by the pineal gland [67]. Melatonin acts as an antioxidant to protect cells, and it exerts chronobiotic functions [22]. Moreover, melatonin is an important free radical scavenger that suppresses deleterious oxidant activities and contributes to the control of the redox state of cells [68].

Induced extrapineal synthesis of melatonin does not contribute to pineal diurnal rhythms [67]. Extrapineal synthesis of melatonin is responsible for the circulation of the endocrine and paracrine-produced melatonin [67]. Several neurotransmitters, including glutamate, acetylcholine, vasoactive intestinal peptide, substance P, and pituitary adenylate cyclase-activating peptide are associated with direct central modulation of the melatonin synthesis process [21]. Melatonin synthesized in the gastrointestinal tract in a nonrhythmic manner has a protective role in the gastric mucosa against stress conditions [69].

With regard to one research, the regulatory role of melatonin contributes the immune defense response [70]. In addition, the synthesis of melatonin by extrapineal tissues has been clearly shown to be related to defense responses, such as activation of polymorphonuclear and mononuclear cells in the blood [71], peritoneum [72], and colostrum [73]. The immune-competent cells, such as phagocytes, are activated to produce melatonin upon injury stimuli [74, 75]. Activated mononuclear and polymorphonuclear cells synthesize melatonin, and subsequently contribute to the recovery process by suppressing oxidative stress and by boosting macrophage phagocytic activity [73]. In summary, immune cells could produce melatonin in blood and this melatonin could regulate immune responses against stress condition.

Innate immune responses require the recruitment of leukocytes to the site of the lesion. Moreover, the maintenance of circulating leukocytes and the termination of migration during the resolution of the immune response is an important process [76]. Melatonin contributes to the regulation of leukocyte migration to injury sites [76, 77]. Therefore, melatonin is able to control immune responses by inhibiting the activation of inflammatory signaling and by regulating the proliferation and activation of immune competent cells [78, 79]. Recent study reported that melatonin synthesized by macrophages and microglia could suppress their immune activity and increase their phagocytic capacity, characteristics associated with an anti-inflammatory, the M2-like phenotype [80]. As mentioned, melatonin may be an important anti- inflammatory mediator in inflammatory responses and immune actions.

Furthermore, melatonin inhibits the activation of nuclear factor kappa B (NF-κB) as a key transcription factor that mediates the inflammatory response [81]. In the central nervous system (CNS), NF-κB is associated with both innate and acquired immune responses, and it is necessary for neuronal survival [82]. NF-κB modulates neurite outgrowth [83], determines cell fate [84], circuit formation, and brain tissue homeostasis [85]. In the pineal gland, NF-κB is translocated into the nucleus of cells during the daytime [86]. The nocturnal production of melatonin is regulated by several cytokines that interfere with the NF-κB pathway [67]. Melatonin suppresses NF-κB activation in macrophages [87], T cells [88], and neuronal cells [89].

Additionally, some studies have shown that melatonin plays an anti-inflammatory role that is mediated by the inhibition of NF-κB nuclear translocation [90, 91]. Melatonin boosts the phagocytic activity of mononuclear cells, and the expression of the interleukin 2 (IL-2) through MT1 and MT2 membrane receptors [92]. Considering one study, the promoter of the gene that codes for AANAT includes κB sequences [67]. A current study demonstrated that the immune-pineal axis orchestrates the timing of leukocyte migration and alters monocyte phenotype through the NF-κB pathway [93]. The mechanisms of melatonin synthesis in the rat pineal gland has been known to be related with AANAT activation by regulating pCREB [94, 95], and by activating NF-κB [96]. There are some researches proving that the NF-κB transcription factor could modulate a phosphorylation of CREB in cellular mechanism [97, 98]. According to these evidences, the activation of NF-kB in pinealocytes through immune cells at AD brain results in the activation of *Aanat* gene, leading to the production of melatonin (Fig. 1b).

Melatonin reduces the rolling and adherence of leukocytes and neutrophils to the endothelial layer, decreasing vascular permeability [77, 99]. Melatonin is known to inhibit endothelial nitric oxide synthase activation in blood vessels and, subsequently, reduces vascular inflammation [100]. The adhesiveness of neutrophils to endothelial cells inversely correlates with the melatonin concentration in blood [101]. Endothelial cells decreased the expression of adhesion molecules, including platelet endothelial cell adhesion molecular-1 (PECAM-1) and intercellular cell adhesion molecular-1 (ICAM-1), when melatonin levels were increased at night [101]. One study demonstrated that the reduced expression of pro-inflammatory proteins and the increase in anti-inflammatory proteins, such as CD180, in endothelial cells was caused by the increased melatonin production at night [102]. In terms of chronobiotic pattern, melatonin also has chrobiotic antioxidant and anti-apoptotic properties [103]. Therefore, melatonin also could control the vascular homeostasis related with immune responses.

The melatonin synthesized by both pineal gland and extrapineal glands coordinate with each other to regulate immune molecules, including pathogen-associated molecular patterns (PAMPs), danger-associated molecular patterns (DAMPs), toxic Aβ, heat-shock proteins, and tissue debris [67]. Several studies described this bidirectional communication process between the pineal gland and the immune response as “the immune-pineal axis” [67, 104, 105].

In AD, pineal dysfunction leads to decreased melatonin production [37, 38], suggesting that melatonin production is positively linked to promoting neuroprotection [106, 107]. A current study reported that Aβ was observed to interact with toll like receptor (TLRs) in the pineal gland of AD patients, and that the interaction subsequently triggers the synthesis of pro-inflammatory cytokines, and inhibits the expression of *Aanat* and synthesis of melatonin through the NF-κB pathway [108]. The increase of the pro-inflammatory cytokine tumor necrosis factor (TNF), caused by reduced melatonin production is considered as biomarker of AD progression [109]. In AD, pineal gland calcification leads to reduced total melatonin excretion [54], and the resulting melatonin deficit aggravates the progress of AD [110].

Ultimately, the production of melatonin in immune cells is organically linked to neuropathogenesis organically the melatonin secreted from pineal gland. To sum up, pineal gland dysfunction triggers the reduction of melatonin, and leads to the aggravation of inflammation, the abnormal immune response, and the impairment of vascular homeostasis, involving in the neuropathology in AD.

### Sleep disturbance by pineal gland dysfunction and AD

The effect of sleep in human has been reported to be beneficial in many aspects, such as tissue repair, improvement of memory consolidation, and the preservation of neuroimmune-endocrine integrity [111, 112]. Sleep is a vital phenomenon that is generally divided into two phases, sleep with rapid eye movements (REM) and sleep without rapid eye movements (non-REM) [113]. REM sleep is known to be important in memory function, neurogenesis, and the regulation of blood-brain barrier homeostasis [114], whereas non-REM sleep is associated with the release of diverse hormones and is characterized by decreased blood pressure [115].

Sleep disorders occur in 25–66% of AD patients [116]. Current studies showed that sleep disturbance leads to cognitive decline [117, 118], and increases the risk of AD by increasing Aβ burden. [119, 120]. Previous researches demonstrated that the increase of inflammation in the brain with chronic sleep deprivation could boost the risk of neurodegenerative disease onset [121, 122]. Other studies demonstrated that aggravated inflammation caused by sleep disturbance triggers cognitive decline and promotes the onset of AD [123, 124]. Further, sleep quality in AD patients worsens with AD progression [125]. The association between cognitive decline and impaired sleep quality has been reported in AD models with increased Aβ deposition [126, 127]. One brain positron-emission tomography (PET) study mentioned that sleep impairment was related with increased Aβ load in healthy subjects [128]. Furthermore, several studies found that lower sleep quality was associated with an increased brain Aβ load in normal brains [129, 130].

Several studies suggested that sleep dysfunction worsens AD pathology and increases the risk of developing dementia [119, 131]. Furthermore, a recent study demonstrated that reduced glucose uptake in the hypothalamus leads to sleep impairment and can be used as a CSF biomarker of AD [132]. Another study reported that transgenic amyloid precursor protein/presenilin 1 (APP/PS1) mouse model of AD showed significant hypothalamic abnormalities prior to memory loss [133]. In AD, sleep disturbance is linked to physiological changes in the suprachiasmatic nucleus (SCN) and pineal gland [12]. Considering previous reports, the sleep impairment is commonly observed in AD patients and is regarded as the strong booster related with the aggravation of AD pathologies.

Recently, reduced melatonin due to lower pineal gland volume has been observed in AD brains, establishing the relationship between lower pineal gland volume and cognitive impairment in AD patients [134]. Several studies suggested that reduced pineal volume results in insomnia, and is significantly associated with sleep disturbances in AD [18, 134, 135]. Therefore, the pineal gland dysfunction by lower pineal gland volume contributes directly to the sleep deficit in AD patients. Taken together, the decrease in melatonin secretion due to pineal gland dysfunction triggers insomnia, sleep disturbance, and poor sleep quality, and ultimately, results in memory loss in AD.

### Impaired neurogenesis by pineal gland dysfunction and AD

Neural plasticity is an important feature of brain function, because continuous adaptation in changing conditions is essential to preserve homeostasis [136]. Neurogenesis is a major component of plasticity in response to CNS damage and occurs at sites of brain injury [136]. Neural stem cells (NSCs) are multipotent and are present in the sub-ventricular zone (SVZ) of the forebrain and the subgranular zone (SGZ) of the hippocampus [137]. Neurogenesis in the hippocampus is important for the maintenance and recovery of cognitive function [138], suggesting that the circuitry of the hippocampal dendrite gyrus outputs to the dorsal CA3, is associated with the encoding of time in new memories [139].

The latest study reported that melatonin contributes to structural plasticity in axons of the hippocampal dentate gyrus [140]. Judging from some studies, melatonin induces the proliferation and survival of NSCs in the midbrain and the hippocampus [141, 142]. A couple of studies also reported that melatonin promotes neurogenesis in the hippocampus of C57BL/6 mice [143, 144]. It was also assumed that the melatonin effect on NSCs was mediated by neurotrophic factors, such as brain-derived neurotrophic factor (BDNF) and glial cell-derived neurotrophic factor (GDNF) [145]. In brief, melatonin has a cardinal effect in the improvement of neurogenesis and synaptic plasticity (Fig. 1c).

Recent research demonstrated that the thalamus, including the pineal gland, has emerged as a host site for such a neurogenic niche [146, 147]. A number of studies report that the hypothalamus has the ability for neurogenesis [148, 149]. Latest studies suggest that NSC proliferation and neurogenesis in the thalamus are increased when the day length decreases [150, 151]. It was found that hypothalamic neurogenesis is more activated depending on the day length [147], and neurogenesis is related to daylight and melatonin secretion [151]. NSC proliferation in the SVZ was independent of seasons and was shown to be influenced by reduced melatonin secretion through pinealectomy in animal models of AD [152].

In AD brains, neurogenesis in hippocampal areas is attenuated compared to the normal brains [153]. Decreased neurogenesis and neuronal loss was observed in the hippocampus and cortex areas of AD mice [154, 155], and the positive correlation between memory loss and level of neurogenesis in AD has been reported [155, 156]. Additionally, the impaired neurogenesis in the dentate gyrus of AD mice has been reported by decreased numbers of doublecortin, a new neuronal marker, positive cells [155, 157]. Neurogenesis is impaired by reduced melatonin secretion in AD. Therefore, the pineal gland dysfunction is related with the impaired neurogenesis, leading to memory loss in AD brain.

Ultimately, pineal gland dysfunction, which reduces melatonin secretion, may be one of the crucial factors that causes impaired neurogenesis in AD. Finding the way to improve pineal dysfunction may be a key to circumvent impaired neurogenesis in AD brain.

## Conclusion

The progression and onset of AD is complicated, as the disease is related with other organs and is affected by various factors. Here, I have summarized the recent findings of pineal dysfunction in AD pathogenesis.

I highlight three points in this review (Fig. 1). First, pineal dysfunction involving reduced pineal volume and pineal calcification in AD contributes to the impaired immune-pineal axis. Melatonin secreted from the pineal gland and extrapineal organs are important mediators of communication between pineal gland and the immune system in AD. Second, pineal dysfunction in AD results in reduced melatonin production, which finally triggers sleep disturbance and poor sleep quality in AD. Third, pineal dysfunction in AD is a critical factor in the inhibition of neurogenesis in AD brains. Pineal dysfunction leads to the reduced hippocampal neurogenesis and hypothalamic neurogenesis. Finally, that these alterations due to pineal dysfunction are linked to memory loss in AD.

Recently, there are some interesting approaches to overcome pineal gland dysfunction. Epigenetic trial may be a solution to solve the pineal dysfunction in AD, suggesting from recent study that the function of pineal gland could be epigenetically regulated by valproic acid (VPA) that up-regulates the expression of melatonin receptor in brain [158]. Moreover, the regulation of noncoding RNAs such as miR-325-3p may be a solution to control the secretion of melatonin by modulating the expression of *Aanat* genes in pineal gland, suggesting that the interaction between miR-325-3p and the 3’UTR of *Aanat* mRNA 3′-UTR control the activation of *Aanat* gene expression in pinealocytes [159].

Based on the significant consequences stated above, I highlight the need for the further study of pineal gland function in AD and to find more effective solution to overcome pineal gland dysfunction in AD with various points. Hence, I assume that this review may provide new concepts in understanding the importance of pineal gland function on diverse AD neuropathology.

## Data Availability

Not applicable.

## Abbreviations

AD: Alzheimer’s disease
APP/PS1: Amyloid precursor protein/presenilin 1
Aβ: β-amyloid
BDNF: Brain-derived neurotrophic factor
CNS: Central nervous system
CSF: Cerebrospinal fluid
DAMPs: Danger-associated molecular patterns
GDNF: Glial cell-derived neurotrophic factor
ICAM-1: Intercellular cell adhesion molecular-1
IL-2: Interleukin 2
MT1: Melatonin receptor 1
MT2: Melatonin receptor 2
NFTs: Intracellular neurofibrillary tangles
NF-κB: Nuclear factor kappa B
NSCs: Neural stem cells
PAMPs: Pathogen-associated molecular patterns
PECAM-1: Platelet endothelial cell adhesion molecular-1
REM: Rapid eye movements
SCN: Suprachiasmatic nucleus
SGZ: Subgranular zone
SVZ: Sub-ventricular zone
TLRs: Toll like receptor
